# The impact of postpartum anemia on cognitive function: a study using 3D video game-based assessment of reaction times in women

**DOI:** 10.3389/fpsyg.2025.1598851

**Published:** 2025-11-20

**Authors:** Shao Patricia Cajas-Cerna, José Antonio Portilla-Fernández, Carlos Andrés Mugruza-Vassallo

**Affiliations:** 1 San Juan Bautista Private University, Lima, Peru; 2 University of Dundee, Division of Arts and Science, School of Arts and Science, Dundee, United Kingdom; 3 Grupo de Investigación en Computación y Neurociencia Cognitiva, Universidad Tecnológica de Lima Sur (UNTELS), Lima, Peru

**Keywords:** anemia, postpartum, selective attention, cognitive function, multitasking, ICT, video game, long-term studies

## Abstract

**Objective:**

To determine the visual and risk factors associated with postpartum anemia in women treated at Sergio Bernales Hospital, Peru, in 2022, and to evaluate the long-term cognitive impact of anemia in 2025.

**Methodology:**

An observational, cross-sectional, and correlational study was conducted. Data were collected from 184 medical records of postpartum women. Cognitive function was assessed after nearly three years in 30 postpartum women using a 3D video game based on selective attention and for discussion multitasking paradigms. Statistical analysis included chi-square tests and odds ratios (OR) for medical records, and ANOVA for visual attention.

**Results:**

Clinical factors such as cesarean delivery (OR = 3.320), uterine atony (OR = 6.120), lack of prenatal care (OR = 9.117), obesity (OR = 7.120), short interpregnancy interval (<3 years, OR = 9.720), and preterm gestational age (OR = 4.530) were strongly associated with postpartum anemia. Cognitive testing revealed significant differences in reaction times between women with and without anemia (Game 1: *p* = 0.014; Game 2: *p* < 0.001), indicating a long-term impact on selective attention and multitasking abilities.

**Conclusion:**

Risk factors for postpartum anemia include cesarean delivery, uterine atony, lack of prenatal care, obesity, intergenesic period <3 years, and preterm gestational age. Cognitive testing nearly three years postpartum in 3D videogames showed significant differences in reaction times, suggesting a long-term impact on cognitive health. More studies are needed to study plasticity and long-term anemia impact.

## Introduction

Postpartum anemia is a significant public health issue affecting both mothers and infants globally. According to the World Health Organization (WHO), 26.8% of the global population suffers from anemia, with pregnant women being particularly vulnerable, with a prevalence of 35.5% (World Health Organization, 2025). The prevalence of anemia among postpartum women is alarmingly high in some countries since seminal work of Taylor et al. (1981), with studies indicating rates as high as 69% in Ethiopia (Lakew et al., 2024), 17.8–45.4% in Peru (Hernández-Vásquez et al., 2017), 32.7% in China (Zhao et al., 2019), 29% in Spain (Medina Garrido et al., 2018), and 22% in Germany (Bergmann et al., 2010). This condition can lead to significant maternal complications, including fatigue, cognitive impairment, and increased risk of postpartum depression, while also adversely affecting neonatal outcomes such as low birth weight (Habtamu et al., 2025; Abioye and Fawzi, 2024) as well as developmental delays (Kruger and Méndez, 2021). A systematic review and meta-analysis by Kang et al. (2020) found that anemia is significantly associated with an increased risk of maternal depression (OR/RR: 1.53, 95% CI: 1.32–1.78), highlighting the importance of addressing anemia to improve both physical and mental health outcomes in postpartum women.

Anemia is linked to cognitive impairments due to chronic hypoxia, affecting attention and executive functions (Agrawal et al., 2019). A study found a significant correlation between low hemoglobin levels and decreased cognitive performance, as measured by the Mini-Mental Status Examination (MMSE) (Agrawal et al., 2019). Furthermore, Greig et al. (2013) demonstrated that iron deficiency in women of childbearing age negatively impacts cognition, mental health, and fatigue, with iron supplementation improving cognitive performance in several studies. Also, Lalitha et al. (2024) demonstrated that pregnant women with anemia exhibit significantly longer reaction times (RTs) compared to non-anemic pregnant women, highlighting the direct impact of anemia on cognitive function during pregnancy. These findings underscore the importance of addressing iron deficiency to improve both cognitive and mental health outcomes.

During pregnancy, anemia-induced hypoxia reduces cerebral oxygen delivery in the mature maternal brain, impairing neuronal metabolism and synaptic plasticity, particularly in attention-related prefrontal regions. Animal studies using chronic intermittent hypoxia models in adult rats demonstrate reduced dendritic spine density, altered synaptic ultrastructure, and impaired long-term potentiation (LTP) in the prefrontal cortex and hippocampus, correlating with deficits in attention and working memory (Gozal et al., 2001; Payne et al., 2004; Xie and Yung, 2012). This suggests that hypoxia disrupts synaptic signaling and plasticity in prefrontal cortical neurons not only in newborns (Wang et al., 2021) but also in mothers, potentially contributing to cognitive impairments. However, direct evidence in mothers remains limited.

Although human data are sparse, clinical studies report that anemic pregnant women exhibit slower reaction times (Lalitha et al., 2024) and executive dysfunction consistent with hypoxia-induced prefrontal cortical impairment. Furthermore, anemia during pregnancy is linked to increased oxidative stress and inflammation, which may exacerbate neural dysfunction; prophylactic iron supplementation in nonanemic pregnant women can increase oxidative stress, whereas in anemic women, it improves hematological status and reduces inflammation without worsening oxidative stress (Rajendran et al., 2022).

Moderate hypoxia negatively impacts selective attention, particularly in complex cognitive tasks and gamified cognitive assessments have been proposed as effective tools to measure cognitive function under hypoxic conditions (Hite et al., 2022). Maternal iron levels have been shown to influence infant cognitive outcomes, suggesting a link between maternal health and cognitive function in offspring (Thomas et al., 2017). Furthermore, maternal iron deficiency anemia has been shown to negatively impact postpartum emotional and cognitive functioning, with iron supplementation improving depression, stress, and cognitive performance in affected mothers (Beard et al., 2005).

On the other hand, research indicates that visual stimuli in 3D environments can enhance cognitive assessments, providing insights into reaction times and attention (Kamali-Ardekani et al., 2022; Thomas et al., 2017). Cognitive function, particularly selective attention, plays a crucial role in postpartum recovery. Selective attention refers to the ability to focus on specific stimuli while ignoring others, a process that can be impaired by anemia due to chronic cerebral hypoxia [study case in García et al., 2017]. Selective attention mechanisms are commonly probed using the Posner paradigm, where participants respond to cued (valid/invalid) or uncued targets to measure attentional orienting efficiency (Posner et al., 1980). The neural basis of spatial cognition is deeply tied to environmental geometry. Seminal work by O'Keefe and Burgess (1996) demonstrated that hippocampal place cells encode location through geometric boundaries, creating cognitive maps sensitive to the shape of the environment. This aligns with evidence that humans use visual landmarks and boundary proximity to orient themselves (Hartley et al., 2004). While our 3D video game assesses RTs in a dynamic environment, it shares conceptual overlap with Posner-like attentional demands, particularly in disengaging and shifting focus amid distractors.

Recent studies have explored the impact of visual stimuli on cognitive function, particularly in 3D environments, using video games to measure reaction times (RT) as an indicator of cognitive response(Torres-Tejada et al., 2020). Torres-Tejada et al. (2020) made a video game designed to evaluate and enhance selective attention measures using 3D stimuli presented on a 2D screen. The game leverages the principles of selective attention, where participants process visual stimuli before making decisions. For instance, Takahashi et al. (2024) demonstrated that a 3D action puzzle video game could effectively assess executive functions in children and adolescents with ADHD, providing a convenient and ecologically valid alternative to traditional neuropsychological tests.

The integration of Information and Communication Technologies (ICT) in healthcare has opened new avenues for understanding cognitive impairments. For instance, Mugruza-Vassallo et al. (2022) demonstrated how different Markov chains modulate visual stimuli processing in 2D, 3D, and augmented reality environments, highlighting the potential of ICT in cognitive assessment. Similarly, Mugruza-Vassallo and Miñano-Suarez (2016) emphasized the role of ICT in enhancing productivity and innovation in healthcare, particularly in developing regions like South America. Therefore anemia after pregnancy may impact, not only health, but also society around women.

This study aims to identify the risk factors associated with postpartum anemia and explore the impact of selective attention on cognitive function using 3D video games. By analyzing both clinical and visual factors, this research seeks to provide a comprehensive understanding of postpartum anemia and its long-term cognitive effects.

## Methods

### Study design

This study employed an observational, cross-sectional, and correlational design. Data were collected from 184 medical records of postpartum women treated at Sergio Bernales Hospital in 2022 (ethics under Ethics Certificate 1,208-2023-CIEI-UPSJB). Cognitive function was assessed after nearly three years (ethics under Ethics Certificate 0353-2025-CIEI-UPSJB) using a 3D video game based on selective attention (Torres-Tejada et al., 2020) and multitasking paradigms (Mugruza-Vassallo et al., 2021). Statistical analysis included chi-square tests, odds ratios (OR), and ANOVA.

### Participants

The study population consisted of postpartum women diagnosed with anemia and treated at the Department of Gynecology and Obstetrics at Sergio Bernales Hospital in Peru between February and December 2022. A sample of 184 women was selected using probabilistic random sampling. For cognitive testing, 15 women with anemia and 15 without anemia were assessed in 2025. The Hospital Nacional Sergio Bernales, located in Lima, Peru, serves a population of approximately 2.3 million people, making it a critical center for studying postpartum anemia in the region.

### Data collection

Clinical and gynecological data were collected via standardized forms extracting information from medical records. Variables included sociodemographic factors (age, residence, occupation, education, income), clinical factors (cesarean delivery, uterine atony, prenatal care, parity, body mass index), and obstetric factors (interpregnancy interval, gestational age).

**Figure 1:**
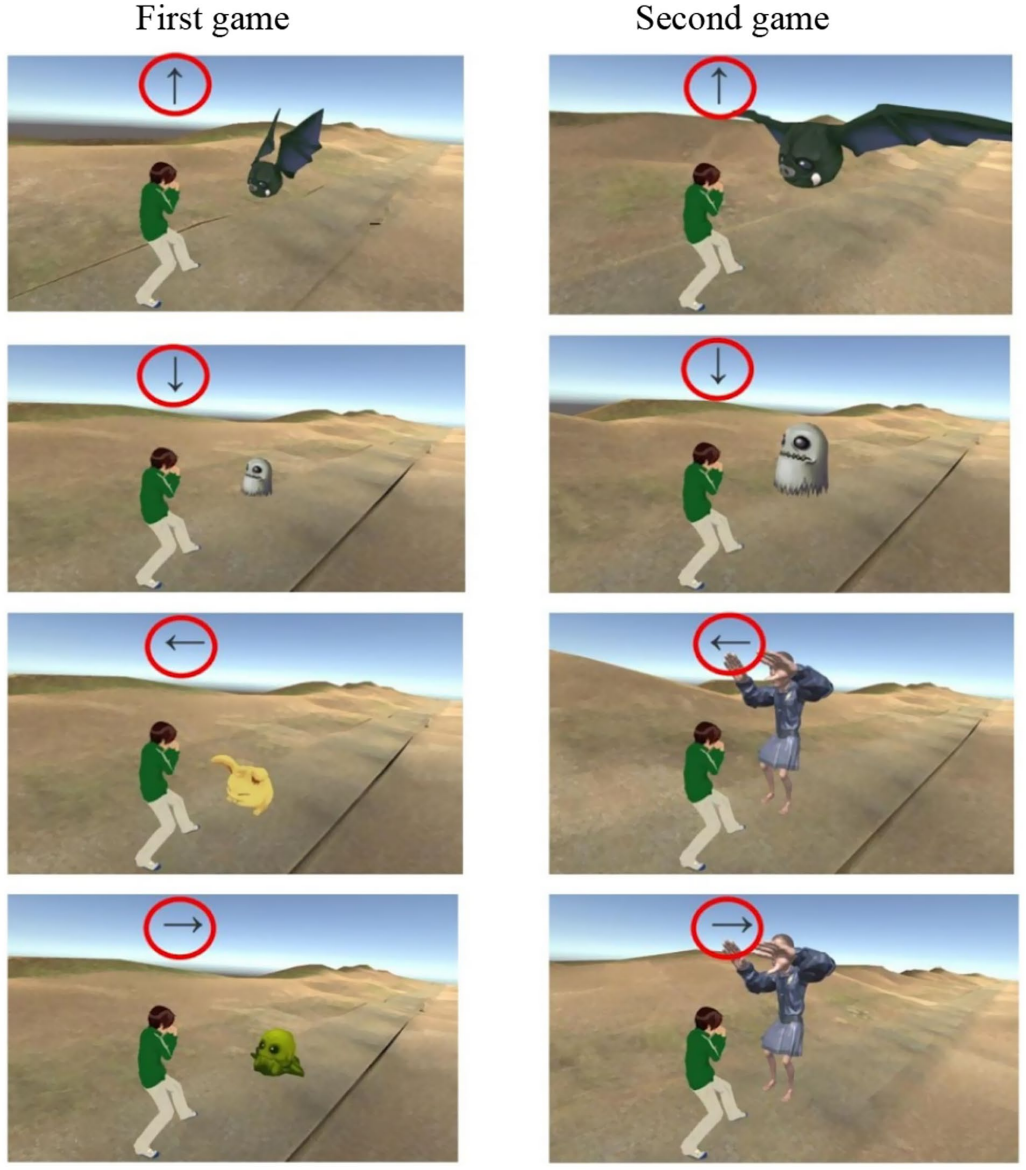
3D video game on a 2D screen for selective attention. Taken and Inspired by the research of Torres-Tejada et al. (2020).

Cognitive function was assessed using a Unity 3D-based 3D video game (Torres-Tejada et al., 2020) using visual warning cues (Posner et al., 1980), which measured reaction times to visual stimuli and multitasking performance (see Figure 1). Two distinct 3D games evaluated cognitive function in participants with and without anemia. Game Design:

First game: Stimulus scaling was derived from a similarity ratio (Equation 1) using a focal point, stimulus height, and distances between the stimulus and focal point. A proportionality rule (Equation 1) was applied to calculate stimulus scaling in Unity.

$$\begin{array}{l} \text{Height}\;(\text{screen}\_\text{stimulus})=\text{distance}\;(\text{focus},\text{target})\\ /d\;{\left(\text{stimulus},\text{focus}\right)}^{*}\;h\;(\text{real}\_\text{stimulus}). \end{array}$$ (1)

Second game: Stimulus sizes were adapted from the original 2D game using direct 2D-to-3D proportionality ratios. Default stimulus sizes were used to determine scaling via a simple proportionality rule in Unity, i.e., stimulus size in the top right of Figure 1 appears bigger than stimulus size in top left.

### Statistical analysis

Descriptive statistics were used to summarize the data. Chi-square tests and odds ratios were used to assess associations between risk factors and postpartum anemia. To compare the reaction times between participants in the “WITH” (“CON”) and “WITHOUT” (“SIN”) anemia groups, a one-way analysis of variance (ANOVA) was conducted. The ANOVA was performed using the f_oneway function from the scipy.stats module in Python. This statistical test was chosen to determine if there were significant differences in reaction times (Tiempo) between the two groups at both videogames, followed by one-way ANOVAs for *post hoc* group comparisons. Assumptions of homogeneity of variance were confirmed via Levene’s test (*p* = 0.646).

A two-way ANOVA (*α* = 0.05) assessed main effects (Game Type and Anemia Condition) and interaction.

Table 1 summarizes the sociodemographic characteristics of postpartum women treated at Sergio Bernales Hospital in 2022, highlighting the prevalence of postpartum anemia across different categories such as age, residence, employment status, education level, and household income.

**Table 1:** Sociodemographic characteristics of postpartum women treated at Sergio Bernales Hospital in 2022.

Characteristic	Postpartum anemia (%)	No anemia (%)
Patient age		
20–30 years	88.9	11.1
30–40 years	85.5	14.5
Patient residence		
Comas	88.2	11.8
Carabayllo	85.4	14.6
Employment during pregnancy		
Worked	86.7	13.3
Did not work	88.6	11.4
Educational level		
Completed primary education	90.3	9.7
Completed secondary education	83.3	16.7
Higher education	85.7	14.3
Approximate monthly household income		
Below minimum wage (<1,000)	89.1	10.9
Above minimum wage (>1,000)	55.6	44.4

## Results

### Risk factors associated with postpartum anemia in women

The analysis identified several significant risk factors for postpartum anemia, as summarized in Table 1. Cesarean delivery was associated with a 3.32-fold increased risk (OR = 3.320, *p* = 0.048), while uterine atony showed a 6.12-fold increased risk (OR = 6.120, *p* = 0.032). Lack of prenatal care had the strongest association, with a 9.12-fold increased risk (OR = 9.117, *p* = 0.004). Obesity (OR = 7.120, *p* = 0.003), an intergenesic period of less than 3 years (OR = 9.720, *p* = 0.025), and preterm gestational age (OR = 4.530, *p* = 0.015) were also significant risk factors (Table 2).

**Table 2:** Clinical and gynecological characteristics of postpartum women with anemia.

		Anemia postpartum *N* (%)	*F* (%)
Clinical characteristics			
Cesarean section				Yes		100	0	No		0	100	Uterine atony				Yes		88.9	11.1	No		85.5	14.5	Prenatal care				Yes		88.9	11.1	No		85.5	14.5	Number of children				1–2 children		90.5	9.5	3–4 children		86	14	First child		84.9	15.1	BMI				Thin		91.8	8.2	Overweight		88.5	11.5	Obesity		81.4	18.6
Gynecological characteristics			
Interpregnancy interval				<3 years		88.9	11.1	>3 years		85.5	14.5	Gestational age				Preterm (<37 weeks)		88.9	11.1	Term (>37 weeks)		85.5	14.5

These findings highlight the multifactorial nature of postpartum anemia and underscore the importance of addressing these risk factors to reduce its prevalence. Interventions such as improved prenatal care, nutritional support, and optimal birth spacing could play a crucial role in reducing the burden of postpartum anemia and its associated complications.

- Cesarean delivery (OR = 3.320, *p* = 0.048).
- Uterine atony (OR = 6.120, *p* = 0.032).
- Lack of prenatal care (OR = 9.117, *p* = 0.004).
- Obesity (OR = 7.120, *p* = 0.003).
- Intergenesic period <3 years (OR = 9.720, *p* = 0.025).
- Preterm gestational age (OR = 4.530, *p* = 0.015).

### 3D visual attention in postpartum anemia on women

#### Homogeneity of variances

Levene’s test did not reject homogeneity of variances (*p* = 0.647), satisfying ANOVA assumptions.

#### ANOVA results for “3D with size 2D with formula_Data”

The results of the one-way ANOVA comparing reaction times between the “CON” and “SIN” groups in the dataset “3D with size 2D with formula_Data” are presented in Table 3. The ANOVA yielded a significant difference in F-statistic of **6.055** and a *p*-value of **0.014**.

**Table 3:** One-way ANOVA for reaction times between postpartum anemia and non-anemia groups in dataset of the 3D videogames in 2025.

ANOVA	Reaction times	
	*F*	*p*
Game 1: 3D with size 2D with formula	6.055	0.014
Game 2: 3D with size 2D without formula	14.503	0.001

#### ANOVA results for “3D with size 2D without formula_Data”

The results of the one-way ANOVA comparing reaction times between the “CON” and “SIN” groups in the dataset “3D with size 2D without formula_Data” are presented in Table 3. The ANOVA yielded a significant difference in F-statistic of **14.503** and a p-value of **0.000145 (*p* < 0.001)**.

#### Two-way ANOVA

Table 4 presents two-way ANOVA results, indicating the anemia condition significantly impacts reaction times in both 3D games (p **< 0.001**), while game type showed no differences (*p* = 0.675). The absence of interaction suggests anemia’s effect is consistent across game versions.

**Table 4:** Two-way ANOVA results for reaction times.

Source of variation	*F*	df	*p*	*η* ^2^
Game type	0.18	1	0.675	0.000
Anemia or no condition	19.35	1	**<0.001**	0.062
Interaction	1.02	1	0.312	0.000

Table 4 presents results from a two-way ANOVA examining the effects of anemia status and game type on reaction times. The analysis revealed a statistically significant main effect of anemia status (*p* < 0.001), indicating that participants with anemia exhibited slower reaction times compared to non-anemic individuals. Conversely, no significant differences were observed between game types (p = 0.675). Notably, the absence of a significant interaction effect (*p* > 0.05) suggests that anemia’s impact on reaction times remained consistent across both 3D game versions.

**Figure 2:**
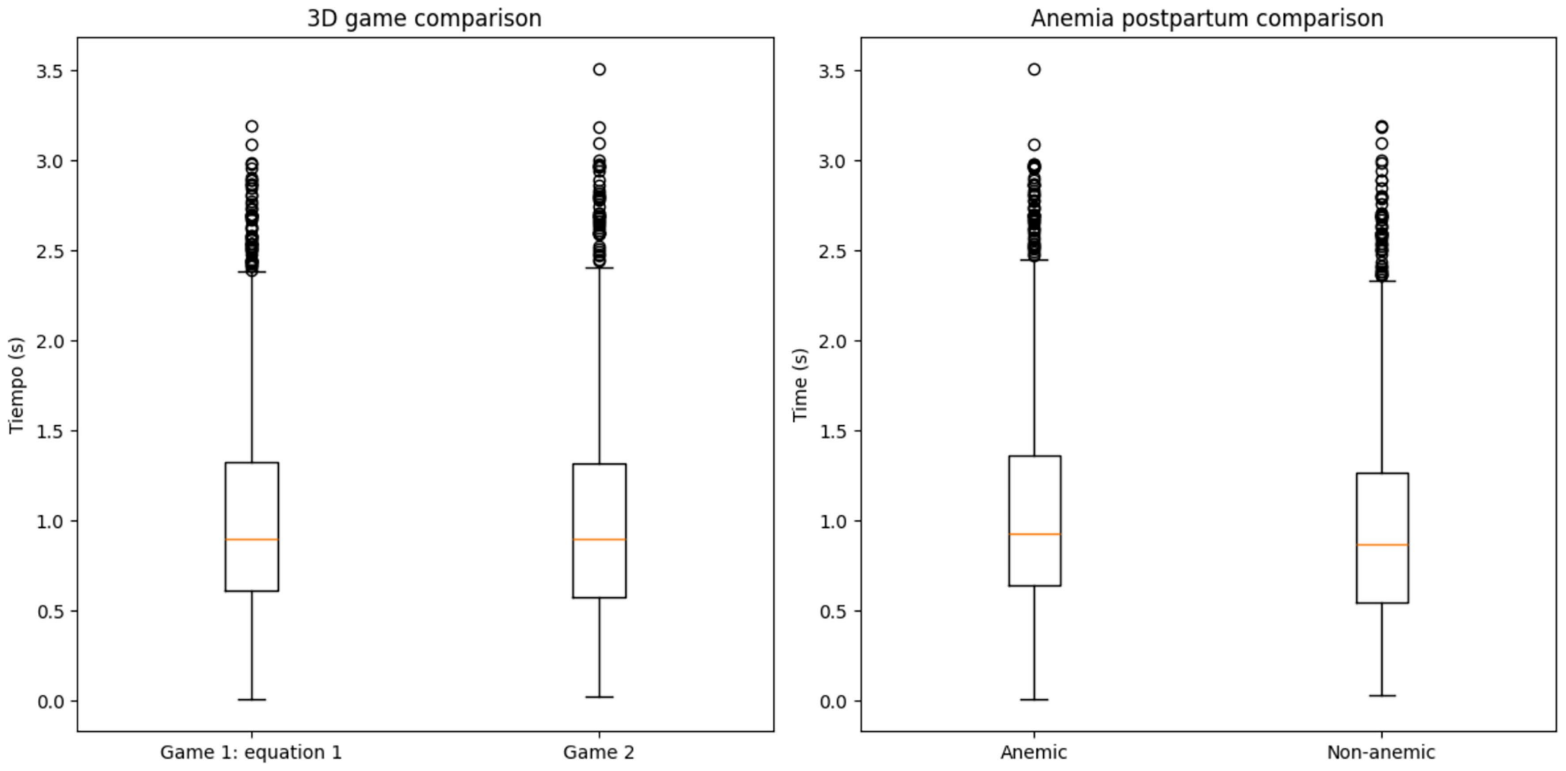
Comparative performance metrics across experimental conditions. (Left panel) Boxplot comparison of completion times between Game 1 (equation-based) and Game 2 (non-equation-based) in 3D gaming scenarios. (Right panel) Boxplot comparison of completion times between anemic and non-anemic participants in postpartum conditions.

Table 4 and Figure 2 collectively present findings from a two-way ANOVA and visual comparisons of performance metrics across experimental conditions.

## Discussion

### On the determinants of the factors for postpartum anemia: clinical and gynecological

The findings highlight the multifactorial nature of postpartum anemia, with cesarean delivery, uterine atony, and lack of prenatal care being significant risk factors. These results align with previous studies, such as Casavilca (2017), who found a strong association between cesarean delivery and postpartum anemia. Recent research by Neef et al. (2024) underscores the importance of early detection and treatment of postpartum anemia, particularly through iron supplementation, which has been shown to significantly improve maternal well-being and outcomes. Our study adds to this body of evidence by demonstrating the long-term cognitive impacts of postpartum anemia, further emphasizing the need for timely intervention.

### Cognitive impairment associated with RTs

Significant differences in reaction times were observed between anemic and non-anemic women in both Game 1 (*p* = 0.014) and Game 2 (p < 0.001). The impact of anemia on cognitive function, particularly selective attention, was evident in the 3D video game experiments. These findings align with Jakobsen et al. (2011), who validated reaction time as a measure of cognitive health, and Lalitha et al. (2024), who demonstrated that pregnant women with anemia exhibit significantly longer RTs compared to non-anemic pregnant women. The consistency of these findings across different populations highlights the importance of addressing anemia to mitigate its cognitive effects.

Furthermore, Takahashi et al. (2024) showed that 3D video games can effectively assess executive functions in children with ADHD, suggesting that such tools have broad applicability for cognitive assessment across different populations and conditions.

The long-term RT deficits observed in anemic women align with growing evidence that anemia disrupts neural plasticity. Hypoxia reduces Brain-derived neurotrophic factor (BDNF) availability and synaptic strength in prefrontal cortex (Duderstadt et al., 2024), potentially impairing the brain’s ability to adapt to postpartum demands. While our study did not measure neurobiological markers, the 3D video game paradigm indirectly probes plasticity-dependent processes (e.g., attentional tuning). Future research combining hemoglobin assays with fMRI or transcranial stimulation could clarify how anemia alters postpartum neuroplasticity.

The slower RTs observed in anemic women may reflect broader disruptions in spatial cognition. Hippocampal place cells encode location relative to environmental geometry (O'Keefe and Burgess, 1996), and human navigation relies on landmark-boundary integration (Hartley et al., 2004). While our task was not explicitly navigational, the 3D stimuli required participants to rapidly localize targets within a bounded virtual space—a process potentially dependent on hippocampal-entorhinal circuits. Anemia-induced hypoxia could impair this spatial-attentional integration, compounding RT delays. Future studies could explicitly test boundary-based attention (e.g., varying target proximity to virtual walls) to dissociate hippocampal contributions.

### The role ICT in cognitive assessment

In addition to selective attention, multitasking abilities are crucial for daily functioning, particularly in the context of information and communication technologies (ICT). The increasing integration of ICT into daily life has heightened the demand for multitasking, which can be particularly challenging for individuals with cognitive impairments.

The integration of ICT in this study, particularly the use of 3D video games, provides a novel approach to assessing cognitive function in postpartum women. This aligns with the work of Mugruza-Vassallo et al. (2022), demonstrated that different Markov chains can modulate visual stimuli processing in various environments differently for women and men showcasing ICT’s potential in cognitive assessments. Furthermore, Takahashi et al. (2024) showed that 3D video games can effectively assess executive functions in children with ADHD, suggesting that such tools have broad applicability for cognitive assessment across different populations and conditions. García et al. (2017) also highlighted the use of augmented reality and 3D environments to provide immersive experiences that enhance cognitive evaluations, particularly beneficial for understanding impairments in older adults.

While the use of a 3D video game provided a novel and ecologically valid approach to assessing cognitive function, it is important to consider the limitations of this methodology in Torres-Tejada et al., 2020, our study extends this approach to postpartum women,

The cognitive consequences of technology dependence extend beyond our 3D video game paradigm. Recent work by Kosmyna et al. (2025) demonstrates that overreliance on AI tools (e.g., ChatGPT for writing) can erode neural engagement and self-monitoring capacity, as evidenced by reduced EEG connectivity and poorer recall in LLM-dependent users. While our study highlights anemia’s biological disruption of attention (e.g., slower RTs), Kosmyna’s findings reveal a behavioral analogue: both chronic anemia and habitual AI use may engender ‘cognitive debt’—where short-term efficiency gains compromise long-term executive function. This parallel underscores the need to contextualize cognitive health within modern technological demands. For postpartum women, whose multitasking burdens are heightened by caregiving, interventions must balance ICT-assisted assessments (like our 3D game) with strategies to sustain endogenous attention resilience.

### Executive function and cognitive performance

The significant differences in reaction times between anemic and non-anemic women (Game 1: *p* = 0.014; Game 2: *p* < 0.001) suggest that postpartum anemia may impair executive function, particularly in tasks requiring selective attention and cognitive flexibility. Executive function encompasses higher-order cognitive processes such as attention, working memory, and the ability to switch between tasks, all of which are critical for effective multitasking and decision-making (Takahashi et al., 2024). The slower reaction times observed in anemic women may reflect deficits in these domains, consistent with prior research linking anemia to impaired cognitive performance (Beard et al., 2005; Agrawal et al., 2019).

Furthermore, the multitasking component of the 3D video game required participants to process multiple stimuli simultaneously, a task that heavily relies on executive function. The poorer performance of anemic women in this task suggests that postpartum anemia may specifically affect the ability to manage competing cognitive demands, a key aspect of executive function. These findings align with studies demonstrating the utility of video games in assessing executive dysfunction in other populations, such as children with ADHD (Takahashi et al., 2024).

The prolonged RTs in anemic women may reflect deficits in attentional disengagement, akin to invalid cue trials in Posner tasks. Future studies could adapt our 3D paradigm to include Posner-like cueing (Posner and Rothbart, 2007), isolating specific attentional subsystems (e.g., orienting, alerting in Fan et al., 2002) affected by postpartum anemia. Also, more conditions in the event-related methods (Mugruza-Vassallo and Potter, 2019) or frequency domain method (wavelet feature) or entropy features in non-linear dynamic method (Guo et al., 2022) may allow to study cognitive load (Mugruza-Vassallo, 2023) in anemia.

### Societal implications and ICT in healthcare

The integration of ICT in healthcare not only addresses individual health issues but also contributes to societal well-being by improving maternal health outcomes and reducing the burden of cognitive impairments on families and communities (Araujo et al., 2024; Nikam and Motwani, 2024). This is particularly relevant given the findings of Neef et al. (2024), who highlight the significant impact of postpartum anemia on maternal physical and mental health, and Greig et al. (2013), who demonstrated that iron deficiency in women of childbearing age negatively impacts cognition, mental health, and fatigue. Additionally, Susič et al. (2023) used machine learning to predict depression and fatigue in postpartum anemia patients, demonstrating the potential of advanced technologies to improve the detection and management of these conditions. Garrido Bustamante et al. (2024) further emphasized the effectiveness of ICT in managing immediate postpartum anemia, highlighting personalized monitoring and early interventions as key strategies. While the benefits of ICT in healthcare are substantial, challenges such as the digital divide and access disparities remain critical issues that need addressing to ensure equitable health outcomes for all women, particularly in developing regions (Nihlani and Rana, 2024).

### Limitations

Despite the significance of our findings, several limitations should be acknowledged. First, although the study initially included 184 women to check typical postpartum population, the final sample consisted of 30 women, which was relatively small, particularly for the cognitive testing component conducted over a 3-year period. Although we made efforts to recruit additional participants, the longitudinal nature of the study posed challenges in retaining a larger cohort. It is important to emphasize that our study targeted a particularly difficult-to-recruit population: postpartum women with anemia and our study is event-related, meaning that small methodological variations can be applied across EEG, fMRI, or eye-tracking studies. This specificity makes our final sample size more notable. Compared to other longitudinal studies with shorter follow-up periods, our retention rate is reasonable. For example, in an not event-related task, Casey (2000) conducted a non-event-related 6-month longitudinal study with three groups—10 women planning pregnancy, 18 first trimester pregnant women, and 24 non-pregnant controls—who completed the study. Similarly, Barda et al. (2021) conducted a 2-years study in 40 pregnant and 40 non-pregnant women. Larger sample sizes are needed in future research to strengthen the generalizability of our findings and reduce heterogeneity.

Second, the study did not standardize the type of medication taken by participants in both groups. Variations in medication use may have influenced cognitive performance and reaction times, introducing potential confounding factors. Future studies should standardize medications or control for their effects when including subjects to ensure more robust and reliable results.

Finally, the 3-year follow-up period, while providing valuable longitudinal data, may have introduced variability due to changes in participants’ health, lifestyle, or environmental factors over time. Also other sensing modalities may be affected by plasticity, as prior studies in blind people (Föcker et al., 2012). Future studies should consider shorter follow-up intervals or more frequent assessments to minimize these effects and capture more precise data on cognitive changes.

## Conclusion

This study identified several significant risk factors for postpartum anemia, including cesarean delivery, uterine atony, and lack of prenatal care. The significant differences in reaction times between anemic and non-anemic women suggest that anemia may have long-term cognitive effects, particularly in tasks requiring selective attention and multitasking. These findings are consistent with Tran et al. (2013), who demonstrated that antenatal iron deficiency anemia and common mental disorders have direct adverse effects on infant cognitive development, further underscoring the importance of early detection and treatment to improve both maternal and child outcomes.

The use of 3D video games offers a promising tool for assessing cognitive function in postpartum women, highlighting the potential of ICT in healthcare. Our findings indicate significant differences in reaction times between the “WITHOUT” (“CON”) and “WITH” (“SIN”) anemia groups, with the most pronounced differences observed in the dataset “3D with size 2D without formula” (*p* < 0.001). These results align with Torres-Tejada et al. (2020), who demonstrated the utility of video games in assessing cognitive function, and extend this approach to postpartum women. However, it is important to consider the limitations of this methodology, as highlighted by Takahashi et al. (2024), who emphasized the need for further validation of video game-based assessments across different populations and settings.

## Data Availability

The datasets presented in this study can be found in online repositories. The names of the repository/repositories and accession number(s) can be found at: https://github.com/cmugruza/Visual_Anemia_Postpartum.
